# Syncope as the Presenting Feature of Splenic Rupture after Colonoscopy

**DOI:** 10.1155/2014/825892

**Published:** 2014-02-20

**Authors:** Daniel Jamorabo, Edward Feller

**Affiliations:** ^1^Alpert Medical School of Brown University, P.O. Box G-9459, 70 Ship Street, Providence, RI 02903, USA; ^2^Department of Health Services, Policy and Practice, Brown University, P.O. Box G-S121, Providence, RI 02912, USA

## Abstract

Splenic rupture is a rare, catastrophic complication of colonoscopy and an exceptional cause of syncope. This injury is believed to be from direct trauma or tension on the splenocolic ligament with subsequent capsule avulsion or else from direct instrument-induced splenic injury. Diagnosis requires a high index of suspicion that may be absent because presentation can be subtle, nonspecific, and delayed anywhere from hours to days and therefore not easily attributed to a recent endoscopy. We describe a case of syncope as the initial manifestation of splenic rupture after colonoscopy. Our patient's pain was delayed; his discomfort was mild and not localized to the left upper quadrant. Clinicians should consider syncope, lightheadedness, and drop in hemoglobin in absence of rectal bleeding following a colonoscopy as possible warning signs of imminent or emergent splenic injury.

## 1. Introduction

Splenic rupture is an exceptional and life-threatening complication of colonoscopy. At least 68 cases of splenic injury following colonoscopy have been documented as of 2009 [1]. Splenic rupture is in turn an unusual cause of syncope, so diagnosis requires a high index of suspicion that may be absent. Presentation can be nonspecific and delayed for days after outpatient discharge and therefore not easily attributed to a recent endoscopy. We describe a case of syncope secondary to splenic rupture after colonoscopy to alert clinicians to consider this rare event as a cause of syncope.

## 2. Case Report

A healthy 63-year-old man underwent an uneventful screening colonoscopy without propofol sedation or procedural difficulty and was asymptomatic when discharged. Three hours later, he became lightheaded and diaphoretic with standing. He had a syncopal episode and hit his head against a wall. He lay down for several hours. When he sat up, lightheadedness and diaphoresis returned; he lost consciousness again. His wife witnessed and confirmed the episode. He was brought to the Emergency Department. He denied postictal confusion or bowel/bladder incontinence. He described mild, poorly localized abdominal discomfort following the second syncopal episode. His medications included amlodipine, simvastatin, and ranitidine for gastro-esophageal reflux.

**Figure 1:**
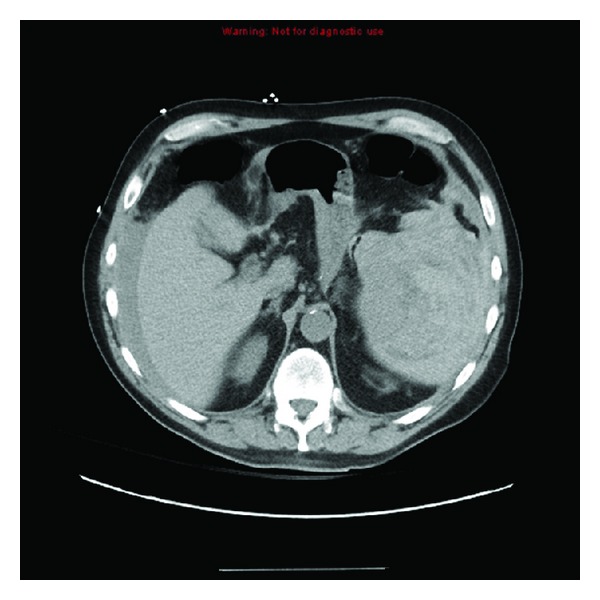
Axial view of splenic rupture and hematoma.

**Figure 2:**
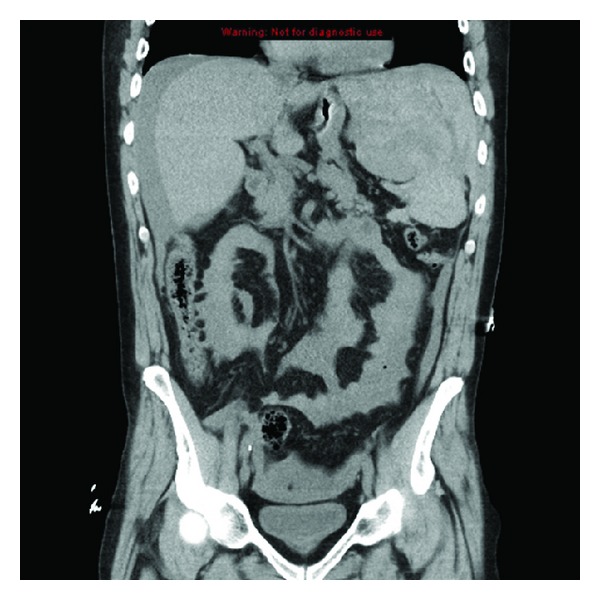
Coronal view. Patient's Hgb had dropped from 12.3 to 8.7 g/dL at this point.

Blood pressure was 127/77 mm Hg on admission without a postural change; the rest of the exam was unremarkable except for mild tenderness to palpation over the left trapezius, diminished bowel sounds, and mild tenderness to palpation in the left lower quadrant without signs of peritoneal irritation. He had a third syncopal event in the Emergency Department two hours after arrival. Systolic blood pressure decreased to the 60s; Hemoglobin dropped from an initial level from 12.3 g/dL to 8.7 g/dL. Abdominal CT scan revealed a 13 × 10 × 13 cm splenic hematoma with splenic rupture (Figures 1 and 2). Head and neck CT was normal. After emergency splenectomy, his hemodynamic status stabilized with no recurrence of syncope.

## 3. Discussion

Splenic injury due to colonoscopy is rare and believed to be due either to direct trauma or to tension on the splenocolic ligament with subsequent capsule avulsion or from direct instrument-induced splenic injury [2]. Risk factors include a pathologic spleen or splenomegaly; anticoagulation; smoking; inflammatory bowel disease; therapeutic or difficult colonoscopy; intra-abdominal adhesions secondary to prior surgery, trauma, or an inflammatory process such as pancreatitis; a tortuous, redundant colon, and propofol sedation, which may predispose to both over-sedation and decreased ability of the patient to respond to pain and provide forewarning of intra-abdominal injury; inadequate bowel preparation limiting visualization; and rapid procedure completion time [3, 4].

Signs of splenic rupture are often subtle and nonspecific, which contributes to delayed diagnosis. The most common symptoms are abdominal pain (93%), usually left-sided, as well as left shoulder pain (Kehr's sign: 88%) [3], which was absent in this patient. Kehr's sign is believed to be due to distention of the splenic capsule or irritation of the left hemi-diaphragm.

Our patient had no abdominal pain until the second syncopal episode, six hours after discharge. His discomfort was mild and not localized to the left upper quadrant. Hemoglobin levels, as in our patient, can be normal at presentation and not reflect bleeding for 6–24 hours after rupture.

Delayed or missed diagnosis is common. Recognition requires a high index of suspicion since a symptom-free interval can last from hours to up to eight days or because abdominal complaints may be absent, mild, or nonspecific and not suggestive of splenic injury. [5, 6] Syncope without abdominal pain, our patient's clinical presentation, is not a typical initial manifestation of splenic rupture. Mild, nonspecific abdominal discomfort due to air insufflation is typical after colonoscopy and may occur in the setting of benign radiographic findings [2]. Our patient's pain was delayed; his discomfort was mild and not localized to the left upper quadrant. Thus, association of syncope with a ruptured spleen was not an easy diagnosis [7] A nonruptured hematoma or walled-off rupture after colonoscopy may occur without suspicious abdominal complaints. Bleeding is intraperitoneal so blood does not enter the GI tract to be detected as overt bleeding hemorrhage.

Contrast-enhanced abdominal CT is vital in diagnosing splenic rupture [3] but was performed only after three syncopal episodes had occurred—almost ten hours after the first episode. Other imaging findings in splenic trauma in addition to rupture include subcapsular hematoma, splenic laceration, perisplenic clot, and hemoperitoneum [1]. Ultrasonography has also been used successfully in some patients. Plain abdominal radiographs are insensitive for demonstrating free air and may delay performance of CT scanning. Selected cases without free rupture into the peritoneum can be observed and treated nonoperatively. Rare case reports describe successful splenic artery embolization [1].

## 4. Conclusions 

Splenic rupture is a rare, dangerous, yet underrecognized complication of colonoscopy. Postdischarge telephone monitoring following colonoscopy with contact at home may detect delayed symptoms. Signs of splenic rupture are often subtle and nonspecific, thereby contributing to delayed diagnosis [1]. Injury can occur in the absence of major trauma or previously diagnosed splenic disease [8]. Clinicians should consider syncope, lightheadedness, and hemoglobin drop without rectal bleeding in the postprocedure period as possible warning signs of pending or emergent splenic injury. Since a symptom-free period of as long as 8 days has been reported, association of symptoms to prior colonoscopy may not be apparent.
